# Supplementary material for: Boosting Broiler Health and Productivity: The Impact of in ovo Probiotics and Early Posthatch Feeding with Bacillus subtilis, Lactobacillus fermentum, and Enterococcus faecium
Source: Microorganisms. 2025 May 27;13(6):1219. doi: 10.3390/microorganisms13061219 (PMC12195048; doi:10.3390/microorganisms13061219)
Supplement: Supplementary file 1 [file microorganisms-13-01219-s001.zip › microorganisms-3594750-supplementary.pdf]

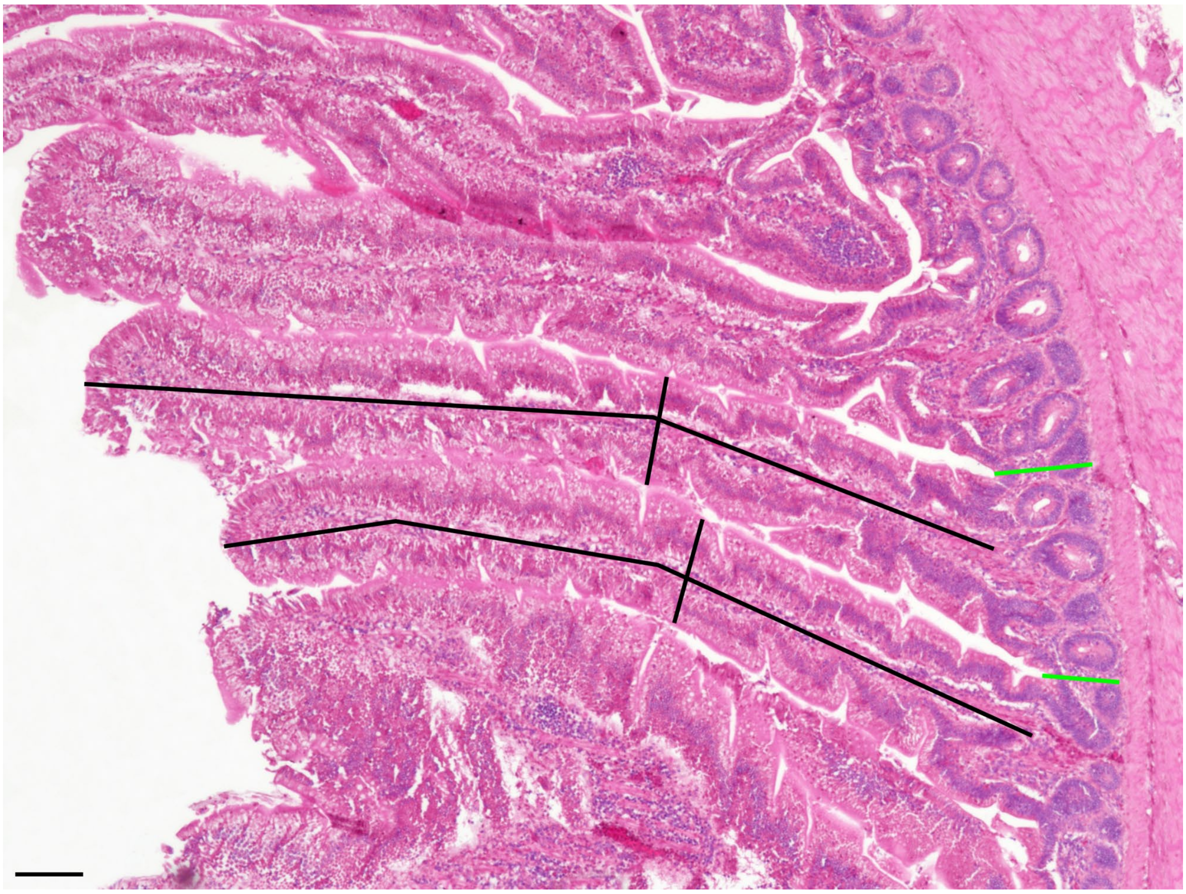

Figure S1. Method of measuring the length and width of villi (black lines) and depth of crypts (green lines) in the intestine. Jejunum, day 35, control, H&E staining, scale bar = 100 μm.
